# Supplementary material for: Hidden magnetism uncovered in a charge ordered bilayer kagome material ScV6Sn6
Source: Nat Commun. 2023 Nov 28;14:7796. doi: 10.1038/s41467-023-43503-9 (PMC10684576; doi:10.1038/s41467-023-43503-9)
Supplement: Supplementary file 1 — Supplementary Information [file 41467_2023_43503_MOESM1_ESM.pdf]

Supplementary Information

Z. Guguchia,[1,*] D.J. Gawryluk,[2,†] S. Shin,[2] Z. Hao,[3] C. Mielke III,[1,4] D. Das,[1] I. Plokhikh,[2] L. Liborio,[5] K. Shenton,[5] Y. Hu,[6] V. Sazgari,[1] M. Medarde,[7] H. Deng,[3] Y. Cai,[8] C. Chen,[8] Y. Jiang,[9] A. Amato,[1] M. Shi,[6] M.Z. Hasan,[9,10,11] J.-X. Yin,[3] R. Khasanov,[1] E. Pomjakushina,[2] and H. Luetkens[1]

[1] *Laboratory for Muon Spin Spectroscopy, Paul Scherrer Institute, CH-5232 Villigen PSI, Switzerland*
[2] *Laboratory for Multiscale Materials Experiments, Paul Scherrer Institut, 5232, Villigen PSI, Switzerland*
[3] *Department of Physics, Southern University of Science and Technology, Shenzhen, Guangdong, 518055, China*
[4] *Physik-Institut, Universität Zürich, Winterthurerstrasse 190, CH-8057 Zürich, Switzerland*
[5] *Scientific Computing Department, Science & Technology Facilities Council, Rutherford Appleton Laboratory, Didcot OX11 0QX, United Kingdom*
[6] *Photon Science Division, Paul Scherrer Institut, CH-5232 Villigen PSI, Switzerland*
[7] *Laboratory for Multiscale Materials Experiments, Paul Scherrer Institut, CH-5232 Villigen PSI, Switzerland*
[8] *Shenzhen Institute for Quantum Science and Engineering, Southern University of Science and Technology, Shenzhen 518055, China*
[9] *Laboratory for Topological Quantum Matter and Advanced Spectroscopy (B7), Department of Physics, Princeton University, Princeton, New Jersey 08544, USA*
[10] *Princeton Institute for the Science and Technology of Materials, Princeton University, Princeton, New Jersey 08540, USA*
[11] *Quantum Science Center, Oak Ridge, Tennessee 37831, USA*

I. SUPPLEMENTARY NOTE 1: LABORATORY X-RAY DIFFRACTION EXPERIMENTS ON A SINGLE CRYSTAL OF SCV_6SN_6

A single crystal of ScV_6Sn_6, obtained from Sn flux, was mounted on the *MiTeGen MicroMounts* loop and used for a X-ray structure determination. Measurements were performed at RT on a *STOE STADIVARI* diffractometer equipped with a *Dectris EIGER* 1*M* 2*R CdTe* detector and with an *Anton Paar Primux* 50 Ag/Mo dual-source using Ag K_α radiation (λ = 0.56083 Å) from a micro-focus X-ray source and coupled with an *Oxford Instruments Cryostream* 800 jet. The unit cell constants and an orientation matrix for data collection were obtained from a least-squares refinement of the setting angles of 16647 reflections in the range $6.7° < 2\theta < 66.6°$. A total of 3448 frames were collected using ω scans, 5 seconds exposure time and a rotation angle of 0.5° per frame, and a crystal-to-detector distance of 60.0 mm.

Data reduction was performed with *X-Area* package [*X-Area* package, Version 2.1, STOE and Cie GmbH, Darmstadt, Germany, 2022]. The intensities were corrected for Lorentz and polarization effects, and an empirical absorption correction using spherical harmonics was applied [X-Area package, Version 2.1, STOE and Cie GmbH, Darmstadt, Germany, 2022]. The structure was solved using *ShelXT* [1] and *Olexs*^{2} program [2]. The model was refined with *ShelXL* package [3] and *Olexs*^{2} software. The data collection and refinement parameters are given in Supplementary Table 1.

II. SUPPLEMENTARY NOTE 2: FITTING PARAMETERS OF THE MAGNETORESISTANCE CURVES

Supplementary Figure 1 depicts the temperature dependences of the parameters n, α and β for ScV_6Sn_6, obtained from the fitting of the magnetoresistance curves using the polynomial function: $\Delta\rho/\rho_{H=0} = \alpha + \beta(\mu_0 H)^n$. All three parameters show the change in the slope across the charge order transition temperature $T^* \simeq 80$ K.

III. SUPPLEMENTARY NOTE 3: MAGNETIZATION AND TRANSPORT EXPERIMENTS

Supplementary Figure 2a and b shows the temperature dependence of the macroscopic magnetization and the electrical resistivity, respectively, for ScV_6Sn_6. Both quantities show the abrupt drop at $T^* \simeq 80$ K, which comes

from the charge ordering below this temperature.

We note that the results presented in the main manuscript is obtained on the crystals, exhibiting the charge ordering temperature at $T^* \simeq 80$ K. This is slightly lower than the one reported in the original publication. The high-quality of the crystal with $T^* \simeq 80$ K was assessed using single-crystal X-ray diffraction (crystal structure) and X-ray fluorescence (composition). The X-ray single-crystal diffraction measurement were done using hard X-ray source (AgK alfa, lambda = $0.56\mathring{A}$) to mitigate the effect of absorption. The whole sphere of reflection was measured down to very good resolution in direct space ($0.5\mathring{A}$) to disentangle all possible features of the crystal structure. We detect no impurity elements; the crystal structure is ordered and stoichiometric according to the results of the refinement. We checked possible deviations from the stoichiometry by refining occupancies; they all appeared to be within 3σ from unity. We also checked that the difference Fourier maps are featureless. In sum, minor deviation of properties between samples with $T^* \simeq 80$ K and $T^* \simeq 90$ K samples could stem from minor differences in microstructure undetectable using the employed probes. What we can say based on the X-ray diffraction measurements that the sample with $T^* \simeq 90$ K is less stoichiometric than the sample with $T^* \simeq 80$ K. This is the reason why we performed muon-spin rotation experiments on the $T^* \simeq 80$ K sample.

To further elaborate on the comparison between samples with $T^* \simeq 80$ K and $T^* \simeq 90$ K, we carried out magnetoresistance measurements also for the $T^* \simeq 90$ K sample and the results are shown in the Supplementary Figure 3 and 4. Absolute value (see the Supplementary Figure 3) as well as the shape of $\rho(H)$ at various temperatures (see Supplementary Figure 4a and b) is very identical for the two samples. This means that despite the differences in T^*, the physics of low-temperature charge ordered state remains the same for both samples.

IV. SUPPLEMENTARY NOTE 4: DIRECTION OF THE INTERNAL FIELD AT THE MUON SITE

Supplementary Figure 5a-c shows the schematic illustration of the muon spin precession around the internal magnetic field, for two extreme cases: the local internal field $\mu_0 H_{int}$ is perpendicular to c and $\mu_0 H_{int}$ parallel to c. For the internal field direction, shown in the Supplementary Figure 5b, the μSR signal from 1-2 (F-B) detectors exhibits the maximum amplitude and no oscillations will be detected in the 3-4 (L-R) detectors. The opposite will be observed for the configuration shown in the Supplementary Figure 5c. Thus, by evaluating the data from all four detectors one can obtain useful information on the direction of the internal field.

Since we observed an enhanced electronic relaxation below T^* in both Δ_{12} and Δ_{34} we conclude that the local field at the muon site cannot lie purely along the c-axis direction (this would lead to an absence of the term Δ_{34}). However, any orientation of the local field which has a significant component in the basal plane is consistent with our data.

V. SUPPLEMENTARY NOTE 5: ANALYSIS OF HIGH-FIELD μSR DATA

In the whole investigated temperature range, two-component signals were observed, as can be seen in the probability field distribution profile (see the Supplementary Figure 6): a signal with fast relaxation $\simeq 0.428(3)$ μs^{-1} (broad signal on the left side of the Fourier spectrum) and another one with a slow relaxation $\simeq 0.05$ μs^{-1} (narrow signal). The narrow signal arises mostly from the muons stopping in the silver sample holder and its position is a precise measure of the value of the applied magnetic field. Fits were made by assuming the separation of the TF-μSR response of the sample (S) and the background (BG) components:

$$A(t) = A_{0,S} P_S(t) + A_{0,BG} P_{BG}(t) \tag{1}$$

Here $A_{0,S}$ and $A_{0,BG}$ are the initial asymmetries, while $P_S(t)$ and $P_{BG}(t)$ are the time evolution of the muon-spin polarizations of the sample and the background component, respectively. The sample and background contributions were described using the Gaussian distribution functions:

$$P_S(t) = \cos(\gamma_\mu B_{\rm int} t + \phi_i)\, e^{-\sigma_S^2 t^2/2} \tag{2}$$

$$P_{BG}(t) = \cos(\gamma_\mu B_{\rm ext} t + \phi_i)\, e^{-\sigma_{BG}^2 t^2/2} \tag{3}$$

Here B_{int} and B_{ext} are the internal and external fields, determined as the mean values of the field distribution from the sample and the silver sample holder, respectively. ϕ_i is the initial phase of the muon-spin ensemble, $\gamma_\mu = 2\pi$ 135.5 MHz/T is the muon gyromagnetic ratio, and σ_S and σ_{BG} are the Gaussian relaxation rates of the broad (sample) and narrow components (silver), respectively.

VI. SUPPLEMENTARY NOTE 6: KNIGHT SHIFT VERSUS MACROSCOPIC SUSCEPTIBILITY

In general, the Knight shift K_{exp} is due to the paramagnetism of the host material, and is therefore closely related to its bulk susceptibility χ. In some simple cases, χ and the Knight shift are linearly related: $K_{exp} = A\chi$, where A is a coupling constant. Then if χ depends on temperature, a plot of $K(T)$ versus $\chi(T)$, with temperature T as implicit parameter (the so-called Clogston-Jaccarino plot), is a straight line with zero intercept.

In the Supplementary Figure 7, we show the temperature dependence of the Knight shift K_{exp} (local susceptibility) for ScV_6Sn_6, measured under the c-axis magnetic fields of $\mu_0 H$ = 2 T, 4 T, 6 T, and 8 T and the temperature dependence of the macroscopic magnetization, measured in the c-axis magnetic field of 2 T. As it is clear from Figure S5, both the local susceptibility and the macroscopic susceptibility shows decrease at the charge order transition temperature $T^* \simeq 80$ K, followed by an increase at lower temperatures. However, the increase of K_{exp} occurs below 30 K while magnetization shows an increase below 70 K. So, while overall temperature dependence between K_{exp} and M looks qualitatively very similar, quantitatively there is a breakdown of the proportionality of the μ^+ Knight shift to the measured bulk susceptibility.

The Knight shift, shown in the Supplementary Figure 7, is an experimental Knight shift K_{exp} . Due to the irregular shape of the crystals, it is not possible to consider the demagnetization factor and estimate precise magnitude of the Knight shift and its temperature dependence. Therefore, we can not further discuss the comparison between local and bulk susceptibilities.

Supplementary Table 1: summary of the crystallographic, refinement, and data collection information from the refinements to the single crystal X-ray diffraction data.

**Crystallographic Data**	
Crystallised from	Sn
Chemical Formula	ScV_6Sn_6
Formula Weight	1060.17 $\frac{g}{mol}$
Crystal colour, habit	metallic, hexagonal
Crystal dimensions [mm]	0.06 × 0.07 × 0.02
Temperature [K]	293(2)
Crystal system	hexagonal
Space group	P6/mmm (191)
Z	1
Reflections for cell determination	13137
2θ range for cell determination [°]	3.5 - 59.8
Unit cell parameters:	
a	5.4739(3) Å
b	5.4739(3) Å
c	9.1988(7) Å
α	90°
β	90°
γ	120°
Cell volume V	238.70(3) Å^3
$F(000)$	459
D_x [g cm^{-3}]	7.393
μ(Ag K_α) [mm^{-1}]	11.265
Scan type	ω
2θ(max) [°]	66.7
Transmission factors (min; max)	0.120; 0.160
Total reflections measured	16893
Symmetry independent reflections	436
R_{int}	0.025
Reflections with $I > 4\sigma(I)$	374
Reflections used in refinement	16647
Parameters refined; restraints	15; 0
Final $R(F)$ [$I > 2\sigma(I)$ reflections]	0.0109
$\omega R(F^2)$ (all data)	0.0263
Weights	$\omega = [\sigma^2(F_o{}^2) + (0.0122P)^2 + 0.2139P]^{-1}$ where $P = (F_o{}^2 + 2F_c{}^2)/3$
Goodness of fit	1.066
Final Δ_{max}/σ	0.000
$\Delta\rho$ (max; min) [e Å^{-3}]	3.1; -3.1

* Electronic address: zurab.guguchia@psi.ch
† Electronic address: dariusz.gawryluk@psi.ch

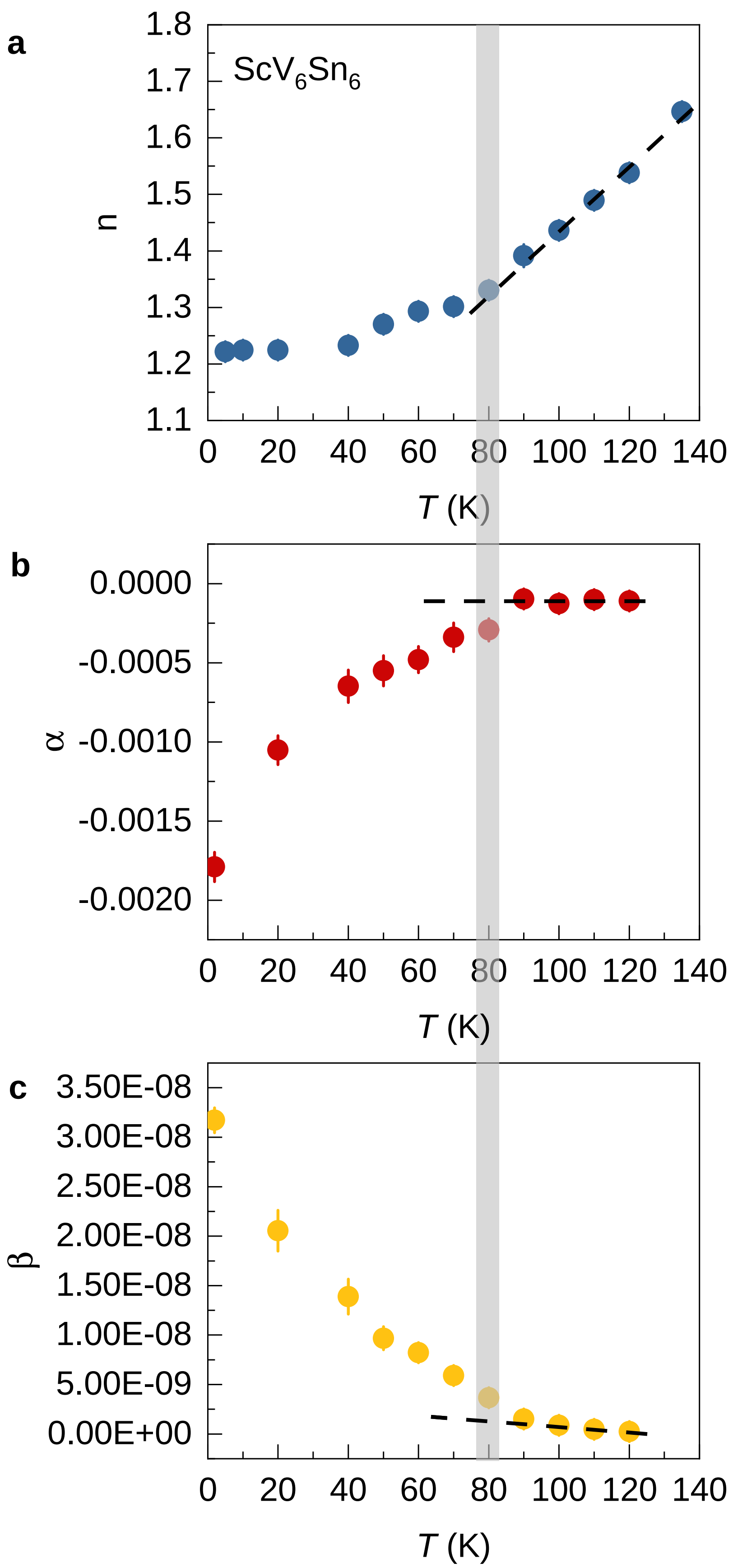

Supplementary Figure 1: **Fitting parameters of the magnetoresistance curves.** The temperature dependences of the parameters n, α and β for ScV6Sn6, obtained from the fitting of the magnetoresistance curves using the polynomial function: $\Delta\rho/\rho_{H=0} = \alpha + \beta(\mu_0 H)^n$. Vertical grey line marks the charge order temperature with $T^* \simeq 80$ K.

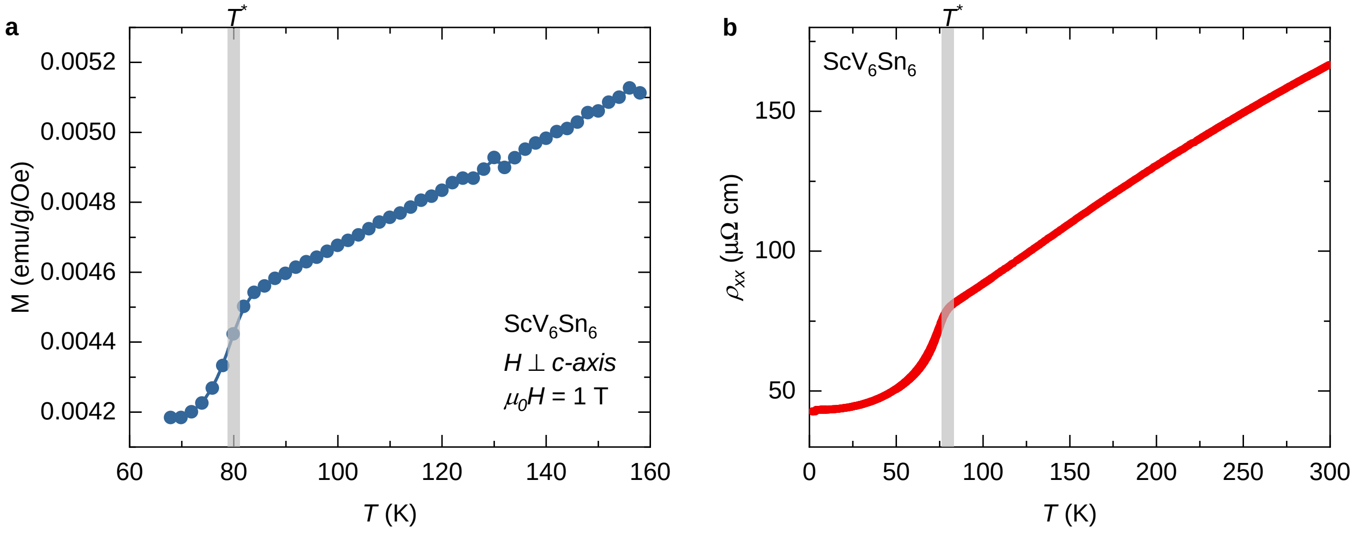

Supplementary Figure 2: **Bulk magnetization and resistivity for ScV_6Sn_6.** The temperature dependence of magnetization (**a**) and resistivity (**b**) for the ScV_6Sn_6 sample. Vertical grey line marks the charge order transition temperature $T^* \simeq 80$ K.

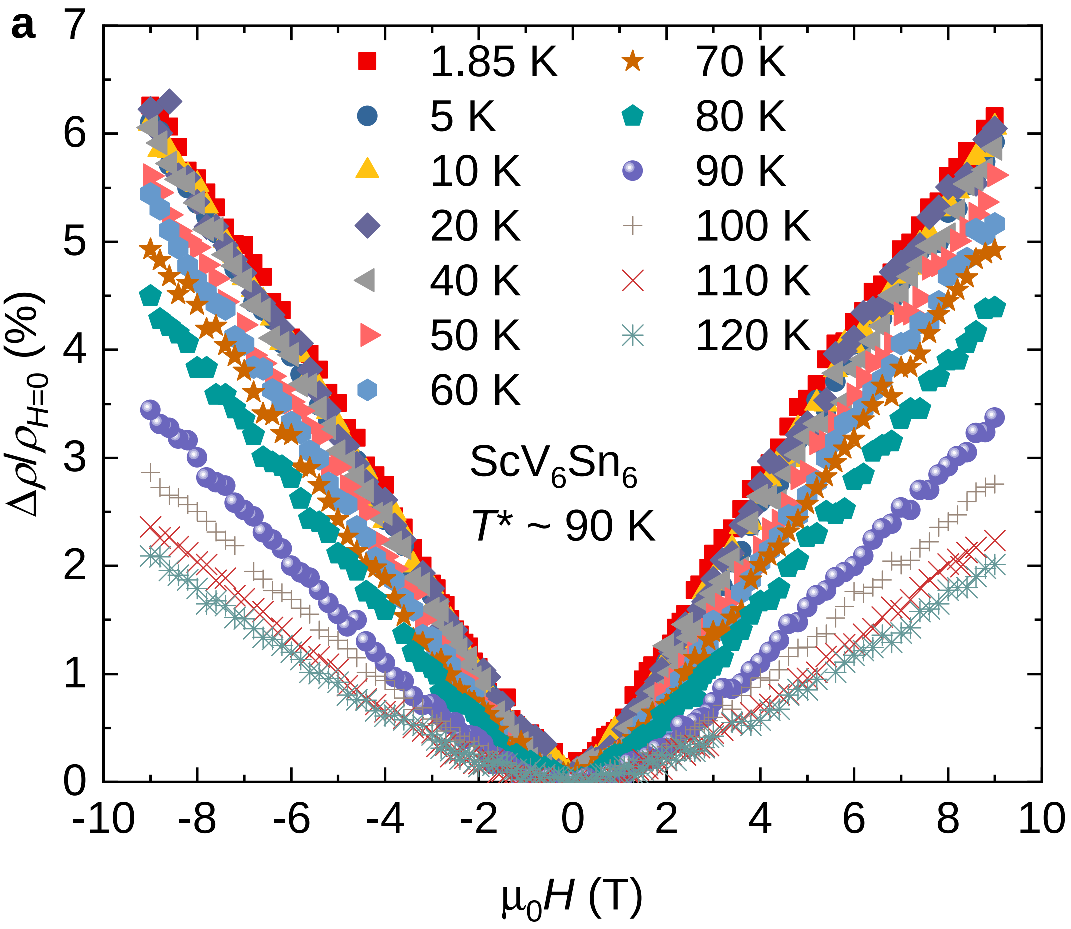

Supplementary Figure 3: **Magnetotransport characteristics of ScV_6Sn_6.** The magnetoresistance for the sample with T^* $\simeq 90$ K, measured at various temperatures above and below the charge ordering temperature.

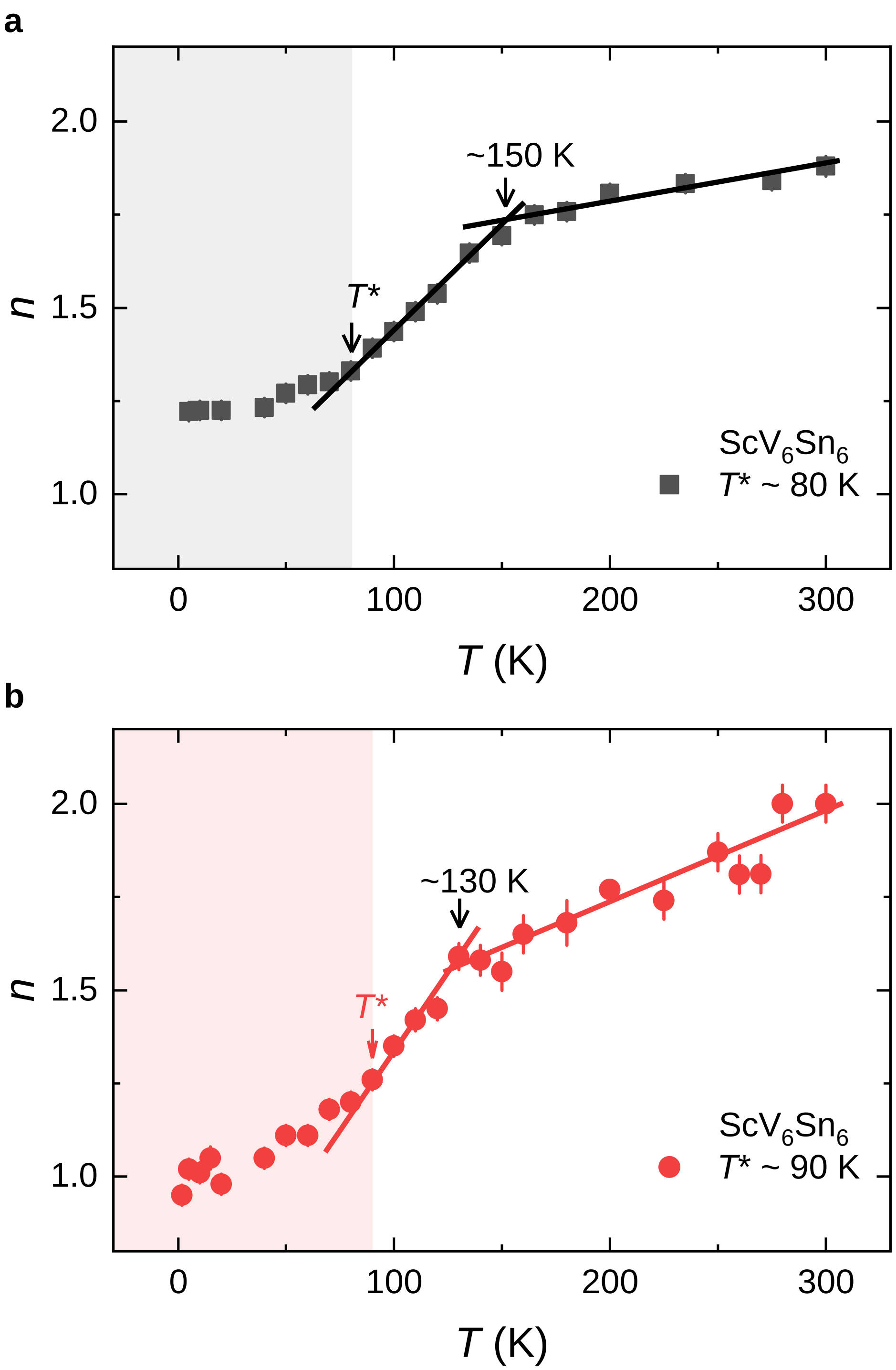

Supplementary Figure 4: **Fitting parameters of the magnetoresistance curves or two samples.** The temperature dependence of the parameter n for the samples with $T^* \simeq 80$ K (**a**) and $T^* \simeq 90$ K (**b**), obtained from the fitting of the magnetoresistance curves using the polynomial function: $\Delta\rho/\rho_{H=0} = \alpha + \beta(\mu_0 H)^n$.

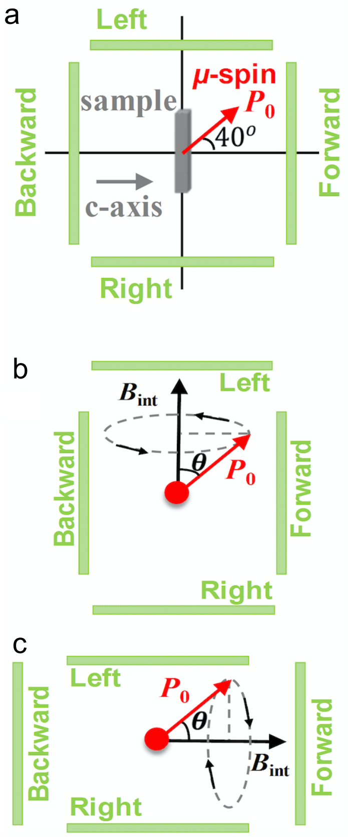

Supplementary Figure 5: **A schematic overview of the zero-field μSR experimental setup. a** A schematic overview of the experimental setup for the muon spin forming 45° with respect to the *c*-axis of the crystal. The sample was surrounded by four detectors: Forward (F), Backward (B), Left (L) and Right (R). **b** – **c** Schematic illustration of the muon spin precession around the internal magnetic field for two cases: **b** The field is perpendicular to the *c*-axis and points towards the L-detector. θ is the angle between the magnetic field and the muon spin polarization at $t = 0$. **c** The field is parallel to the c-axis of the crystal and points towards the F-detector. Adapted from Ref. [4]

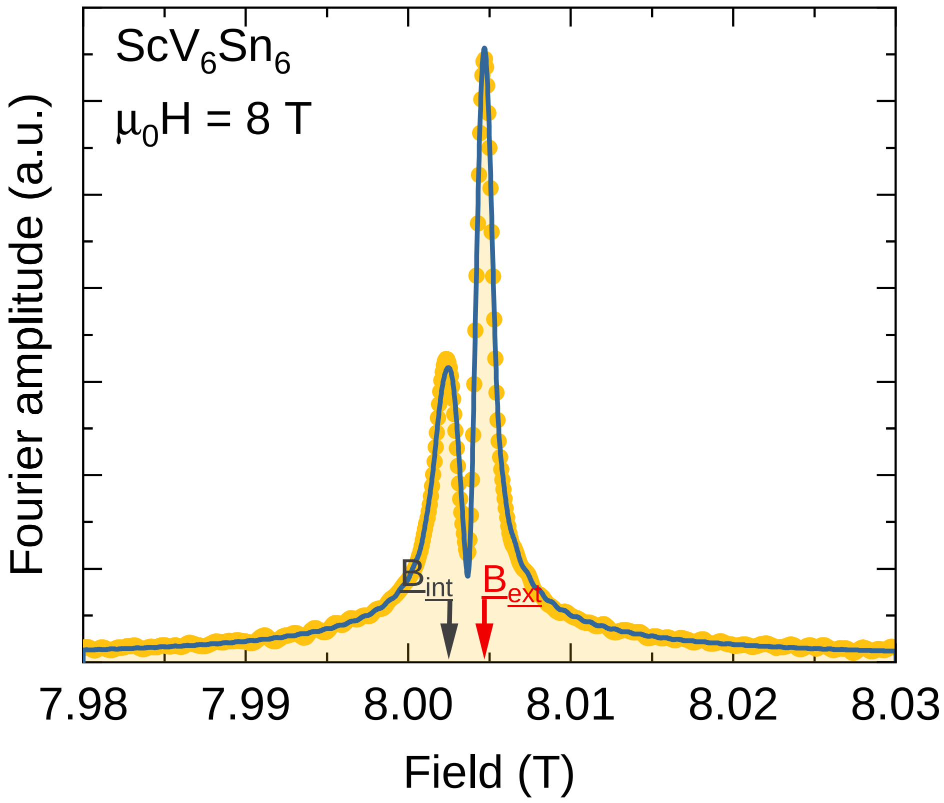

Supplementary Figure 6: **Probability field distribution, measured under high transverse magnetic field. a**, Fourier transform of the μSR asymmetry spectra for a mosaic of single crystals of ScV_6Sn_6 at 3 K in the presence of an applied field of $\mu_0 H = 8$T. The solid line is a two-component signal fit. The peaks marked by the arrows denote the external and internal fields, determined as the mean values of the field distribution from the silver sample holder and from the sample, respectively.

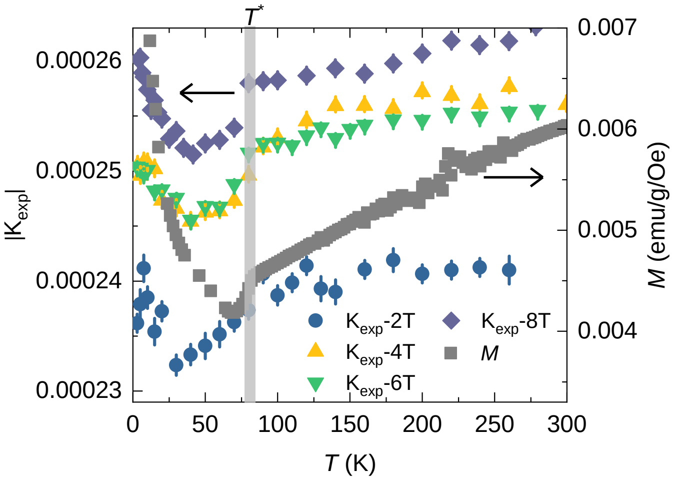

Supplementary Figure 7: **Comparison of Knight shift and macroscopic susceptibility for ScV_6Sn_6.** (Left axis) The temperature dependence of the Knight shift K_{exp} (local susceptibility) for ScV_6Sn_6, measured under the c-axis magnetic fields of $\mu_0 H = 2$ T, 4 T, 6 T, and 8 T. (Right axis) The temperature dependence of the macroscopic magnetization, measured in the c-axis magnetic field of 2 T.

[1] Sheldrick, G.M. SHELXT - Integrated space-group and crystal-structure determination. Acta Cryst. **A71**, 3-8 (2015).
[2] Dolomanov, O.V. et al. OLEX2: a complete structure solution, refinement and analysis program. J. Appl. Cryst. **42**, 339-341 (2009).
[3] Sheldrick, G.M. Crystal structure refinement with SHELXL. Acta Cryst. **C71**, 3-8 (2015).
[4] Guguchia, Z. *et al*. Using uniaxial stress to probe the relationship between competing superconducting states in a cuprate with spin-stripe order. *Phys. Rev. Lett.* **125**, 097005 (2020).
